# EUDOR-A multi-centre research program: A naturalistic, European Multi-centre Clinical study of EDOR Test in adult patients with primary depression

**DOI:** 10.1186/s12888-017-1246-x

**Published:** 2017-03-23

**Authors:** Marco Sarchiapone, Miriam Iosue, Vladimir Carli, Mario Amore, Enrique Baca-Garcia, Anil Batra, Doina Cosman, Philippe Courtet, Guido Di Sciascio, Ricardo Gusmao, Tadeusz Parnowski, Peter Pestality, Pilar Saiz, Johannes Thome, Anders Tingström, Marcin Wojnar, Patrizia Zeppegno, Lars-Håkan Thorell

**Affiliations:** 1 Department of Medicine and Health Sciences, University of Molise, Campobasso, Italy; 2 National Institute for Health, Migration and Poverty (NIHMP), Rome, Italy; 3 National Centre for Suicide Research and Prevention of Mental lll-Health (NASP), Karolinska Institutet, Solna, Sweden; 4 Clinica Psichiatrica, DINOGMI, University of Genoa, Genoa, Italy; 5 Department of Psychiatry, Fundacion Jimenez Diaz University Hospital, Autonomous University of Madrid, Madrid, Spain; 6 Department of Psychiatry and Psychotherapy, University Hospital of Tuebingen, Tuebingen, Germany; 7 Clinical Psychology and Mental Health Department, Iuliu Hatieganu University of Medicine and Pharmacy, Cluj-Napoca, Romania; 8 Department of psychiatry and medical psychology, University Hospital of Montpellier, Montpellier, France; 9 Department of Medical Basic Sciences, Neuroscience and Sense Organs, University of Bari, Bari, Italy; 10 Centro Hospitalar de LisboaOcidental (CHLO), Lisbon, Portugal; 11 2nd Psychiatric Department, Institute of Psychiatry and Neurology, Warsaw, Poland; 12 National Institute of Psychiatry and Addictions, Budapest, Hungary; 13 Department of Psychiatry, University of Oviedo, Oviedo, Spain; 14 Klinikfür Psychiatrie und Psychotherapie der Universität Rostock, Rostock, Germany; 15 Psychiatric Neuromodulation Unit, Department of Clinical Sciences, Faculty of Medicine, Lund University, Lund, Sweden; 16 Psychiatric Neuromodulation Unit (PNU), Department of Clinical Neuroscienses, Malmö University Hospital, Malmö, Sweden; 17 Department of Psychiatry, First Faculty of Medicine, Warsaw Medical University, Warsaw, Poland; 18 Department of Translational Medicine, Azienda Ospedaliero Universitaria Maggiore della Carità, University of Piemonte Orientale “Amedeo Avogadro”, Novara, Italy; 19 EMOTRA AB, Sävedalen, Sweden; 20 Linköping University, Linköping, Sweden

**Keywords:** Electrodermal hyporeactivity, Skin conductance, Intentional self-harm, Suicidal behaviour, Suicide, Depression

## Abstract

**Background:**

Electrodermal reactivity has been successfully used as indicator of interest, curiosity as well as depressive states. The measured reactivity depends on the quantity of sweat secreted by those eccrine sweat glands that are located in the hypodermis of palmar and plantar regions. Electrodermal hyporeactive individuals are those who show an unusual rapid habituation to identical non-significant stimuli. Previous findings suggested that electrodermal hyporeactivity has a high sensitivity and a high specificity for suicide. The aims of the present study are to test the effectiveness and the usefulness of the EDOR (ElectroDermal Orienting Reactivity) Test as a support in the suicide risk assessment of depressed patients and to assess the predictive value of electrodermal hyporeactivity, measured through the EDOR Test, for suicide and suicide attempt in adult patients with a primary diagnosis of depression.

**Methods and design:**

1573 patients with a primary diagnosis of depression, whether currently depressed or in remission, have been recruited at 15 centres in 9 different European countries. Depressive symptomatology was evaluated through the Montgomery-Asberg Depression Scale. Previous suicide attempts were registered and the suicide intent of the worst attempt was rated according to the first eight items of the Beck Suicide Intent Scale. The suicide risk was also assessed according to rules and traditions at the centre. The EDOR Test was finally performed. During the EDOR Test, two fingers are put on gold electrodes and direct current of 0.5 V is passed through the epidermis of the fingers according to standards. A moderately strong tone is presented through headphones now and then during the test. The electrodermal responses to the stimuli represent an increase in the conductance due to the increased number of filled sweat ducts that act as conductors through the electrically highly resistant epidermis. Each patient is followed up for one year in order to assess the occurrence of intentional self-harm.

**Discussion:**

Based on previous studies, expected results would be that patients realizing a suicide attempt with a strong intent or committing suicide should be electrodermally hyporeactive in most cases and non-hyporeactive patients should show only few indications of death intent or suicides.

**Trial registration:**

The German Clinical Trials Register, DRKS00010082. Registered May 31^st^, 2016. Retrospectively registered.

## Background

According to the latest WHO Report [1], every year more than 800,000 people die by suicide. Suicidal behaviour is one of the most common and serious psychiatric emergency [2] and, for this reason, suicide risk assessment is among the most important tasks of mental health professionals.

Nevertheless, little progress has been made towards creating effective suicide risk assessment tools. Indeed, conventional approaches to suicide risk assessment rely on patient self-reporting thus implying several limitations concerning the possible denial of suicidal thoughts [3, 4]. In a recent systematic review [5], no scales for self-harm risk assessment performed sufficiently well so as to be recommended for routine clinical use. However, computer-based tools using implicit association tests showed more promising results [4, 6].

Electrodermal activity (EDA) refers to changes in the electrical conductance of the skin due to sweat gland activity. The reactions depend on the quantity of sweat secreted by those eccrine sweat glands that are located in the hypodermis of palmar and plantar regions. These glands generate sweat excreted through sweating ducts [7]. This sympathetic nerve activity in the skin is regulated not only by environmental temperature, but also by central activations related to affective and cognitive states [8]. Since sweating variations are sensitive markers of events having a particular signification for the individuals, eccrine sweating is also known as palmar, mental or emotional sweating [9], in opposition to the thermoregulatory sweating. For this reason, in research, EDA has been successfully used as indicator of emotional states, such as joy, sadness and fear. In addition, electrodermal reactions are evoked by information processing, such as problem solving, orienting behaviour and learning [10].

EDA comprises tonic and phasic components. Tonic component refers to the slower acting activities and background characteristics of the signal (skin conductance level - SCL), while the phasic component is related to the faster changing elements of the signal, usually associated to a stimulus (skin conductance response - SCR) [11, 12].

**Fig. 1:**
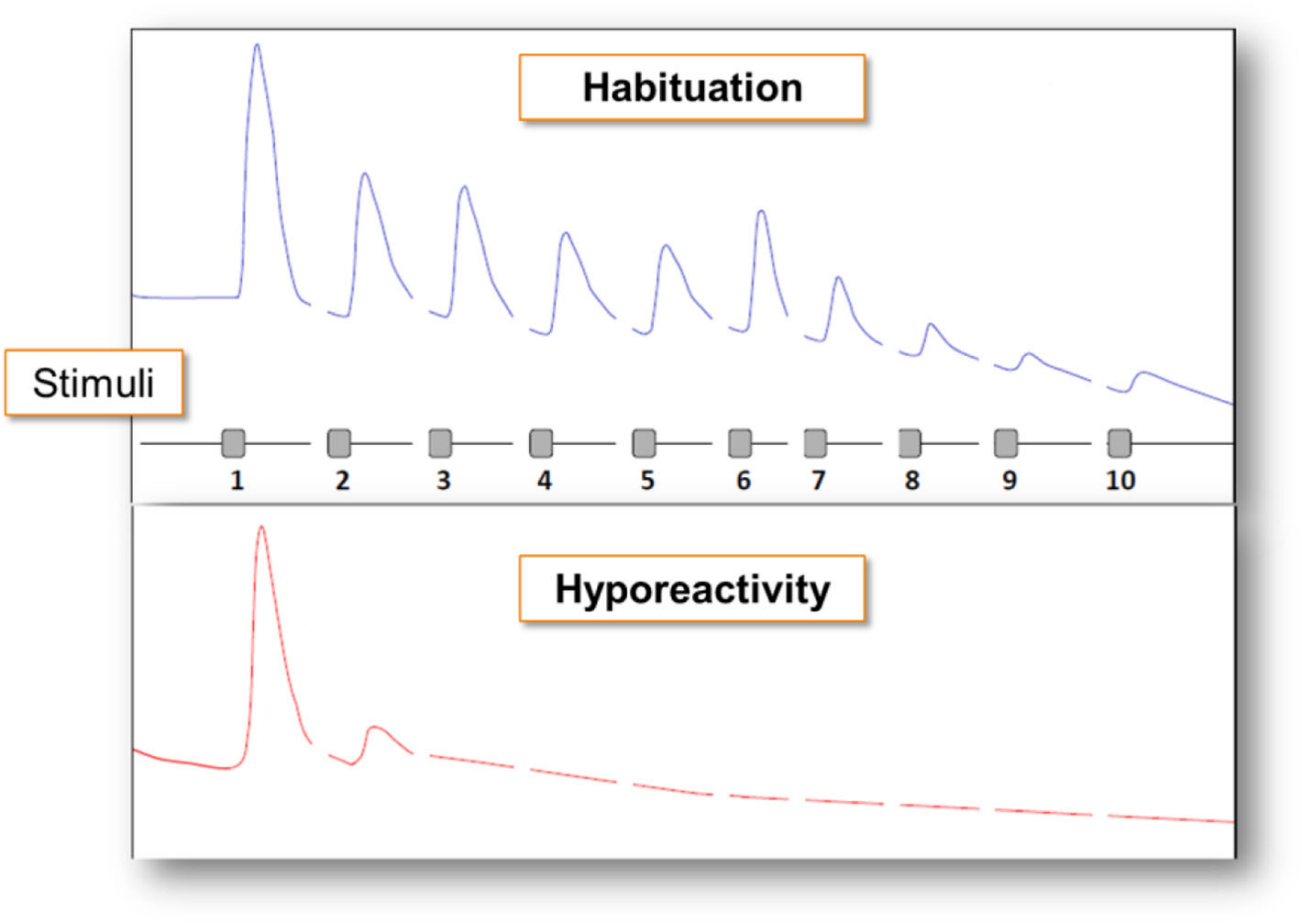
Ideal normal habituation and electrodermal hyporeactivity in relation to the presentation of repeatedly identical non-significant stimuli

The presentation of repeatedly identical non-significant stimuli usually elicits less and less electrodermal reactions, a phenomenon known as habituation [11] or “learning the usual”. Electrodermally hyporeactive individuals are those who show an unusual rapid habituation (Fig. 1). This unusual rapid habituation is supposed to be related to a loss of the normal emotional and information processing reaction of interest to prosaic changes in the environment.

Several studies reported an association between electrodermal hyporeactivity and suicidal tendencies, particularly among depressed patients [13, 14].

The first observations of the relationship between electrodermal hyporeactivity and suicide attempts were made in 1986 by Edman et al. [15]. They found that violent suicide attempters and suicide completers were all fast habituators, whereas the patients with nonviolent methods were equally distributed between slow and fast habituation; on the contrary, suicidal ideators showed the lowest frequency of fast habituators.

Thorell et al. [16, 17] concentrated their studies on depressive patients reporting an extreme electrodermal hyporesponsivity in depressed suicide attempters, but not in non-suicidal depressed patients and healthy controls.

A trend to a faster habituation in suicidal attempters and completers who have used violent methods was also confirmed by Keller et al. [18] and Wolfersdorf & Straub [19]. Differently, Jandl et al. [20] found no difference between violent and non-violent suicide attempters, even if suicide attempters were significantly more hyporeactive than non-attempters.

In a meta-analysis [14] including a sample of 279 depressed patients and 59 healthy subjects, electrodermal hyporeactivity was found significantly associated to high suicide proneness. Indeed, extremely low electrodermal reaction showed a sensitivity of 96.6% and a specificity of 92.9% for suicide and a sensitivity of 83.3% and raw specificity of 92.7% for suicide and/or violent attempt, thus indicating that electrodermal response test may have a high discriminative validity. A further study [21, 22], based on 783 depressive patients, treated at the Weissenau hospital in southern Germany between 1985 and 2002, confirmed previous results and in addition suggested that electrodermal hyporeactivity may be a trait marker for suicidal propensity in depression, independent from its severity, trait anxiety, gender and age.

The neuropsychological basis of electrodermal hyporeactivity seems to lie in the hippocampus. It has been hypothesized that specific neurons in the hippocampus CA3 areas [23] prevent the natural evocation of orienting attention to and curiosity in neutral events in everyday life, which in turn prevents the cognitive and emotional ties to everyday life events. This is thought to lead to preparedness to leave one’s perceived incurious everyday life in face of great difficulties such as depressive thoughts and negative attitudes [24]. Furthermore, this may lead to a loss of fear of impending fear leading to a capability to perform the action [24]. Hyporeactivity is in addition supposed to contribute to a capability to perform the suicidal act with the intention to die [24], due to reduced impulse regulation (difficulty in foreseeing and avoiding strong emotional events), in turn due to a weak input from hippocampus to amygdala [25, 26].

It has been suggested that a suicide related previously unknown neuropsychological dysfunction that prevents the natural eliciting of emotional and cognitive curiosity reactions to events in the everyday life has been detected [27]. The dysfunction is thought to be due to specific impairment of hippocampal functions caused by generally acting biological (e.g., stress, inflammation, genetic influence at many levels) and specific psychological conditions (e.g., learned helplessness, trauma) [27].

A test of ElectroDermal Orienting Reactivity (EDOR Test) specifically optimized for the detection of electrodermal hyporeactivity was developed in the beginning of 1980s [16, 28]. Since then, the test has been constructed in a way that it can be used by any member of the clinical staff at the ward, after a brief training. If confirmed the association between electrodermal hyporeactivity and suicidal tendencies, the EDOR Test could be used as a non-invasive suicide risk screening procedures, which will not require technically skilled personnel or the sophisticated instruments of a psychophysiological laboratory. Indeed, a previous naturalistic study proved that a test of electrodermal hyporeactivity fits very well into the daily clinical work [29].

Emotra AB decided to fund the EUDOR-A study, aimed at investigating the effectiveness and the usefulness of the EDOR Test as an objective support in the suicide risk assessment of patients with a primary diagnosis of depression in the naturalistic setting of psychiatric services. It is hypothesized that the EDOR Test will identify electrodermal hyporeactive depressed patients who in turns will show a higher suicidal proneness as indicated by intentional self-harm behaviours.

Primary objectives of the study are:

- To study the predictive value of electrodermal hyporeactivity measured through the EDOR Test for intentional self-harm (i.e., suicide and suicide attempt), with and without death intent and with and without violent method, in adult patients with a primary diagnosis of depression;
- To gain knowledge about influence from secondary psychiatric diagnoses, from somatic diagnoses, age and gender on this association;
- To study possible traces of influence on the rate of suicide by the clinical use of the EDOR Test.

## Methods/Design

### Study design

EUDOR-A is a naturalistic, non-interventional study performed at 15 different psychiatric centres in 9 different European countries:

- Department of Emergency Psychiatry and Acute Care, University Hospital of Montpellier, Montpellier (France);
- Department of Psychiatry and Psychotherapy, University of Rostock, Rostock (Germany);
- Department of Psychiatry and Psychotherapy, University Hospital of Tuebingen, Tuebingen (Germany);
- III Psychiatric Department, National Institute of Psychiatry and Addictions, Budapest (Hungary);
- Department of Medical Basic Sciences, Neuroscience and Sense Organs, University of Bari, Bari (Italy);
- Clinica Psichiatrica , DINOGMI, University of Genoa, Genoa (Italy);
- Psychiatry Institute, Department of Translational Medicine, University of Eastern Piedmont; S.C. Psichiatria, AOU Maggiore della Carità, Novara (Italy);
- Department of Psychiatry, Medical University of Warsaw, Warsaw (Poland);
- 2nd Psychiatric Department, Institute of Psychiatry and Neurology, Warsaw (Poland);
- Centro Hospitalar de Lisboa Ocidental (CHLO), Lisbon (Portugal);
- Clinical Psychology and Mental Health Department, Iuliu Hatieganu University of Medicine and Pharmacy, Cluj-Napoca (Romania);
- Department of Psychiatry, University of Oviedo CIBERSAM, Oviedo (Spain);
- Department of Psychiatry, Fundacion Jimenez Diaz University Hospital, Autonomous University of Madrid, Madrid (Spain);
- Psychiatric Neuromodulation Unit, Department of Clinical Sciences, Faculty of Medicine, Lund University, Lund (Sweden);
- Psychiatric Neuromodulation Unit (PNU), Department of Clinical Neurosciences, Malmö University Hospital, Malmö (Sweden).

EUDOR-A received the ethical approval at each study site and it was registered in the German Clinical Trials Register (DRKS00010082).

The study planned to recruit at least 1500 patients with a primary diagnosis of depression, also in remission, between August, 2014 and March, 2016. After obtaining the informed consent, a clinical assessment, also including information on the severity of depression and previous actions of intentional self-harm, has been performed. Finally, the patients underwent the EDOR Test to assess their level of electrodermal reactivity. The suicide risk assessment, made during the clinical assessment, could also be revised based on the results of the EDOR Test. Each patient is followed up for one year in order to assess the actions of intentional self-harm occurred in this period.

In the current observational study, the primary variables to be investigated are:

- the prevalence of electrodermal hyporeactivity among depressive patients recruited and tested in the naturalistic setting of psychiatric clinics;
- the incidence of actions of intentional self-harm (i.e., suicide attempt/s before the EDOR Test and during the one-year follow-up period and suicide during the one-year follow-up period).

Secondary outcomes to be assessed are:

- the influence of secondary psychiatric diagnoses, antidepressant treatments, somatic diagnoses, as well as demographic characteristics, such as age and gender, on electrodermal reactivity levels;
- possible traces of influence on the rate of suicide by the clinical use of the EDOR Test.

### Sample size calculation

In order to reach a power of 80% with an alpha level of 0.05 on the Chi-Square test, it has been calculated that at least 47 patients with intentional self-harm (i.e., suicide and suicide attempt) and at least 187 patients without intentional self-harm are required. Increasing the power to 95% at least 78 patients with intentional self-harm (i.e., suicide and suicide attempt) and at least 311 patients without intentional self-harm are required. The study sample potentially exceeds the sample size requirements in order to detect statistically significant changes. This will ensure the required statistical power, taking into account the possibility of some center recruiting fewer pupils than expected, attrition rates at follow-up and missing data.

### Study participants

In- and out-patients with a primary diagnosis of depression, also in remission, have been recruited.

The complete list of allowed primary psychiatric ICD-10 (International Statistical Classification of Diseases and Related Health Problems 10th Revision) [30] diagnoses is showed in Table 1.

**Table 1:** List of allowed primary psychiatric ICD-10 diagnoses

F31 Bipolar affective disorder	F31.3 Bipolar affective disorder, current episode mild or moderate depression
	F31.4 Bipolar affective disorder, current episode severe depression without psychotic symptoms
	F31.5 Bipolar affective disorder, current episode severe depression with psychotic symptoms
	F31.6 Bipolar affective disorder, current episode mixed
	F31.7 Bipolar affective disorder, currently in remission
	F31.8 Other bipolar affective disorders
	F31.9 Bipolar affective disorder, unspecified
F32 Depressive episode	F32.0 Mild depressive episode
	F32.1 Moderate depressive episode
	F32.2 Severe depressive episode without psychotic symptoms
	F32.3 Severe depressive episode with psychotic symptoms
	F32.8 Other depressive episodes
	F32.9 Depressive episode, unspecified
F33 Recurrent depressive disorder	F33 Recurrent depressive disorder
	F33.1 Recurrent depressive disorder, current episode moderate
	F33.2 Recurrent depressive disorder, current episode severe without psychotic symptoms
	F33.3 Recurrent depressive disorder, current episode severe with psychotic symptoms
	F33.4 Recurrent depressive disorder, currently in remission
	F33.8 Other recurrent depressive disorders
	F33.9 Recurrent depressive disorder, unspecified
F34 Persistent mood [affective] disorders	F34.0 Cyclothymia
	F34.1 Dysthymia
	F34.8 Other persistent mood [affective] disorders
	F34.9 Persistent mood [affective] disorder, unspecified
F38 Other mood [affective] disorders	F38.0 Other single mood [affective] disorders
	F38.1 Other recurrent mood [affective] disorders
	F38.8 Other specified mood [affective] disorders
F39 Unspecified mood [affective] disorder	

Patients were eligible to participate only if all the following inclusion criteria were met:

- Primary diagnosis of depression according to the ICD-10, also in remission;
- Age: 18 years or older;
- Gender: Any;
- Signed Informed Consent.

Patients were excluded in case of:

- Dissent to participate in the study;
- Inability to understand the instructions for the EDOR Test;
- Serious problems of hearing.

In cases of diagnosed or suspected dementia or known or suspected alcohol or other substance abuse, the patient was excluded unless there was a special interest by the centre. If included, these conditions were specifically noted.

### Measurements

A comprehensive clinical assessment form has been used in order to collect information concerning socio-demographic characteristics of the subjects, primary and secondary psychiatric and somatic diagnoses, pharmacological and psychological treatment.

Depressive symptomatology has been evaluated through the Montgomery-Asberg Depression Rating Scale [31, 32]. The scale comprises ten items on a 7-point Likert scale (0-6), with higher scores reflecting more severe symptoms of depression. The scale was developed to measure the severity of depressive episodes in patients with mood disorders and explores the following symptoms: apparent sadness, reported sadness, inner tension, reduced sleep, reduced appetite, concentration difficulties, lassitude, inability to feel, pessimistic thoughts and suicidal thoughts.

For the purpose of this study, suicide has been defined as an act that is judged to have been deliberately initiated and performed in the full knowledge or expectation of its fatal outcome [33]. While a suicide attempt represents any action of intentional self-harm with at least some intent to die [33].

Information regarding number, time and method (ICD-10 codes) of previous suicide attempts has been collected. The death intent of the worst attempt concerning death intent, according to the patient, was rated using the first eight items of the Beck Suicide Intent Scale [34]. The original scale is a semi-structured interview including 15 items on a 3-point Likert scale (0-2) assessing the severity of suicide attempts. For the purpose of this study, only the first eight items were used exploring the following characteristics of the suicide attempt: isolation, timing, precautions against discovery/intervention, acting to get help during/after attempt, final acts in anticipation of death, active preparation for attempt, suicide note and overt communication of intent before the attempt. The value of this short version of the Beck Suicide Intent Scale has been previously explored [35, 36].

For each patient, the suicide risk has also been assessed, according to rules and traditions at the centre, and rated on a 5-point scale, form 0 - none to 4 - very high.

**Fig. 2:**
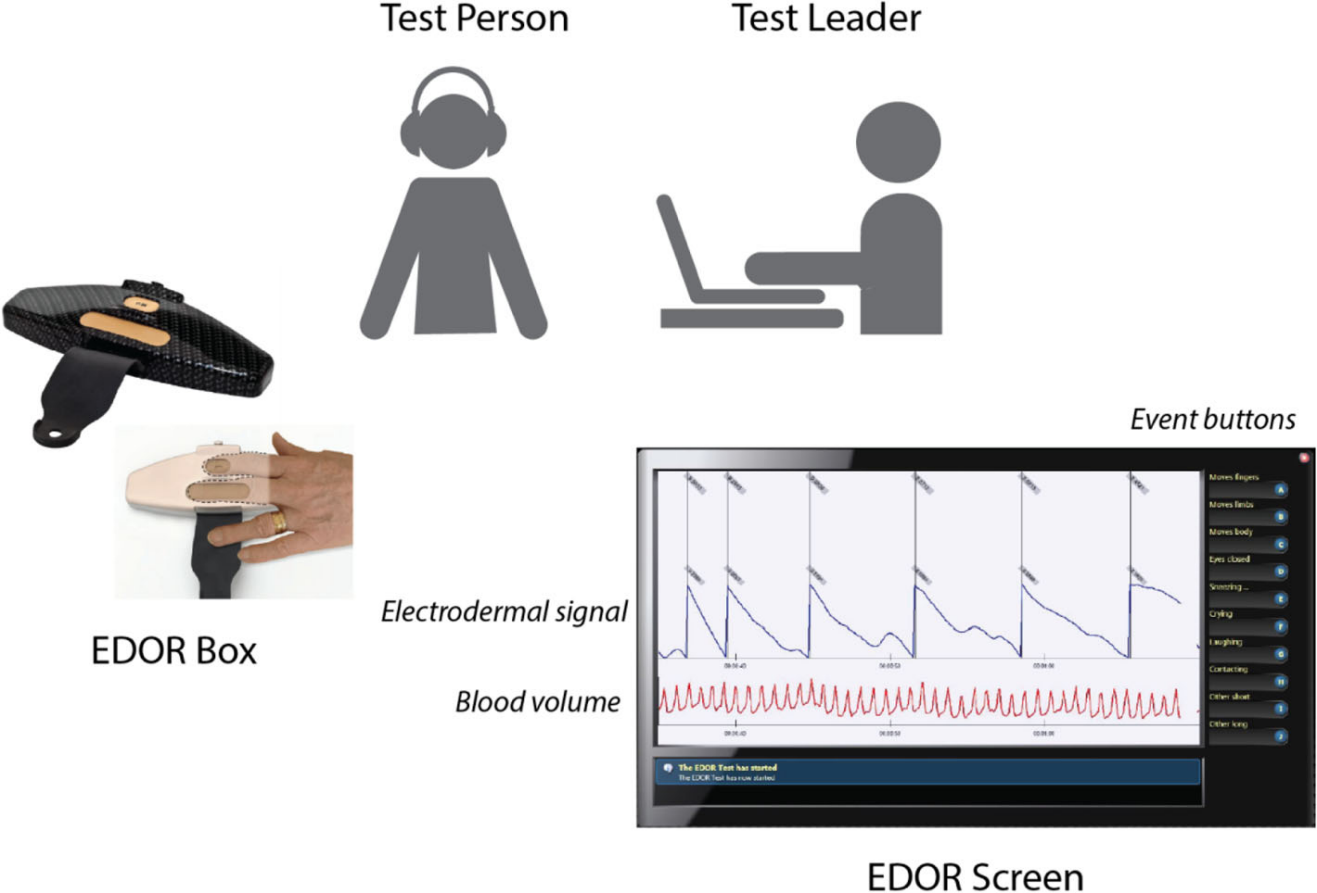
The EDOR (ElectroDermal Orienting Reactivity) Test

After the clinical assessment, the EDOR (ElectroDermal Orienting Reactivity) Test was finally performed by a trained member of the clinical staff (Fig. 2). The EDOR Test is developed by the Swedish company Emotra AB. The test is non-invasive and takes about 15 minutes. During the EDOR Test, two fingers of the non-dominant hand are put on two gold electrodes and a direct current of 0.5 V is passed through the epidermis of the fingers according to standards. A moderately strong tone (1s, 90 dB, 1kHz) is presented through headphones in intervals ranging from 20s – 80s during the test. Sensors located within the electrodes are able to register the electrodermal response to those tones, measuring the skin conductance. The electrodermal responses to the stimuli represent an increase in the conductance due to the increased number of filled sweat ducts that act as conductors through the electrically highly resistant epidermis. Sensors are also able to register blood volume variation.

The EDOR Test is connected via Bluetooth with a computer and the skin conductance signal and the blood volume appear on the screen. Through the events buttons, the test leader can mark certain events having potential significance for the interpretation of the physiological signals (e.g., sneezing, body movements, etc.).

Once the test is finished, data are sent anonymously over the Internet for remote expert analyses at Emotra. Persons who analyse test data are blind to the assessment of clinical data.

Finally, a Test Report is sent by Emotra back to the predefined authorized e-mail of the centre. The test report contains information on the quality of the assessment and the categorization into Hyporeactivity, On the verge of hyporeactivity, Reactivity and Hyperactivity.

Since hyporeactivity was previously found to correlate with suicidal risk [14, 21], based on the results of electrodermal response tests, the clinicians could possible revise the suicide risk assessment.

Each patient is followed up for one year. During the follow-up assessment, data on the number of depressive episodes and actions of intentional self-harm occurred in this period are collected. In particular, information regarding the number of suicide attempts, the method/s used according ICD-10 codes, and if these actions resulted in a suicide is collected. The first eight items of the Beck Suicide Intent Scale are used to evaluate the severity of the death intent in the eventual suicide or worst suicide attempt, according to the patient.

### Statistical analysis

The statistical analyses applied are the computation of the prevalence of Hyporeactivity in the whole and subgroups of the patients, for example socio-economic classifications, groups of diagnoses and medical treatment. This is valid also for the computations of sensitivity and raw specificity (negative predictive value) to types of suicidal behaviours, except for suicide, which has been applied and explained before [17a 17b], which also are computed on the whole material and subgroups. Only non-parametric tests are applied.

### Ethical considerations

The study protocol received the ethical approval from the competent Ethical Committee in each participating centre where the research project has been implemented.

Beside the explanations from their clinicians, the participants received an informational sheet. The participants were informed that the EDOR Test is studied as a new tool for helping doctors in assessing the risk of suicide in depressed patients. They were also informed that the results of the test would be interpreted by an international expert and then transmitted to their doctors.

A signed Informed Consent was obtained for each recruited patient.

In order to ensure confidentiality, a specific code was assigned to each participant and has been used instead of the name in the baseline and follow-up assessments.

Because of the strong evidence today to the favour of tests of electrodermal hyporeactivity in detecting depressive patients with suicidal propensity, it was considered unethical to do a blind study with a control group whose test results were ignored in the clinical assessment of suicide risk. Therefore, each centre was free and strongly encouraged to use the results from the EDOR Test in their clinical suicide risk assessments and planning of the care of the participating patients.

## Discussion

Several studies have reported data showing a strong association between electrodermal hyporeactivity and suicidal behaviour in depressed patients [14, 21]. The findings have promoted the development of the EDOR Test by Emotra AB, Sweden. The test is optimized for detecting hyporeactivity and for routine use by specifically trained staff members of psychiatric clinics.

It is hypothesized that the EDOR Test will identify electrodermal hyporeactive depressed patients who in turns will show a higher suicidal proneness as indicated by intentional self-harm behaviours. Patients that in a suicide attempt reveals strong death intent should be electrodermally hyporeactive in most cases and non-hyporeactive patients should show only few indications of death intent.

It is expected that the test of electrodermal hyporeactivity detects a previously unknown neuropsychological dysfunction that is independent of the depressive state and can be useful in the assessment of suicidality with a high sensitivity and specificity.

The estimated cost for the EDOR equipment is 5500€ and the cost for each analysis 100€. A preliminary economic feasibility study has been performed in the region Östergötland of Sweden (unpublished data). It has been estimated that testing all depressed patients in the region would procure savings for healthcare of 16 million € for a 50% reduction in the number of suicide attempts and 25 million Euro for a 75% reduction in suicide attempts. This estimation does not take into account savings related to suicide preventive strategies for patients with no risk nor the social and psychological costs related to suicide survivors.”

EUDOR-A presents several strengths. It is the first study evaluating the effectiveness and the usefulness of an objective and non-invasive tool (the EDOR Test) in order to be used as support in the suicide risk assessment of depressed patients. Moreover, EUDOR-A is conducted in the naturalistic setting of psychiatric services, so allowing a more easy translation of research findings into the clinical practice. All the members of the staff involved in the research were specifically trained on the use of the EDOR Test, as well as on the collection of clinical information, in order to maximise the reliability and validity of gathered data.

The main limitations of the study rely in the fact that many of the suicide-related information are self-reported. However, the participating centres were strongly encouraged to gather the access also to medical records. Moreover, for ethical reasons it was not possible to conduct a blind study in which EDOR test results were ignored in the clinical assessment of suicide risk. The consequent implementation of suicide preventive interventions and treatments for those patients considered at risk may influence the incidence of suicide attempts in the one-year follow-up period, thus possibly reducing the association between hyporeactivity and suicide proneness.

In conclusion, EUDOR-A is expected to provide scientific evidence for understanding the link between hyporeactivity, as measured by the EDOR Test, and suicidal behaviour in depressed patients, also investigating potential influences exerted by other variables such as secondary psychiatric diagnoses, antidepressant treatments, somatic diagnoses, age and gender. The results of the present study may validate the use of the EDOR Test as an objective and non-invasive tool in the suicide risk assessment of depressed patients.

## Abbreviations

EDA: Electrodermal activity
EDOR Test: ElectroDermal Orienting Reactivity Test
ICD-10: International Statistical Classification of Diseases and Related Health Problems 10th Revision
